# A novel approach for evaluating the effects of odor stimulation on dynamic cardiorespiratory functions

**DOI:** 10.1371/journal.pone.0172841

**Published:** 2017-03-03

**Authors:** Eriko Kawai, Hidehiro Nakahara, Shin-ya Ueda, Kou Manabe, Tadayoshi Miyamoto

**Affiliations:** Graduate School of Health Sciences, Morinomiya University of Medical Sciences, Osaka City, Osaka, Japan; Duke University, UNITED STATES

## Abstract

We aimed to develop a novel method to quantitatively evaluate the effects of odor stimulation on cardiorespiratory functions over time, and to examine the potential usefulness of clinical aromatherapy. Eighteen subjects participated. Nine people were assigned to each of the two resting protocols. Protocol 1: After resting for 2 min in a sitting position breathing room air, the subject inhaled either air or air containing sweet marjoram essential oil from the Douglas bag for 6 min, Protocol 2: After resting for 5 min in a supine position, the subject inhaled the essential oil for 10 min, and then recovered for 10 min breathing room air. All subjects inhaled the essential oil through a face mask attached to one-way valve, and beat-to-beat heart rate (HR) and arterial blood pressure (BP) as well as breath-by-breath respiratory variables were continuously recorded. In both protocols, during fragrance inhalation of the essential oil, time-dependent decrease in mean BP and HR were observed (P<0.05). During post-inhalation recovery, the significant fragrance-induced bradycardic effect lasted at least 5 min (- 3.1 ± 3.9% vs. pre-inhalation baseline value, p<0.05). The mean BP response at the start of odor stimulation was approximated by a first-order exponential model. However, such fragrance-induced changes were not observed in the respiratory variables. We established a novel approach to quantitatively and accurately evaluate the effects of quantitative odor stimulation on dynamic cardiorespiratory functions, and the duration of the effect. This methodological approach may be useful for scientific evaluation of aromatherapy as an approach to integrated medicine, and the mechanisms of action of physiological effects in fragrance compounds.

## Introduction

Inhalation of some fragrances has been used to correct sympathetic dysfunction associated with various lifestyle-related disorders such as hypertension and obesity [1–4]. Furthermore, several studies found that the essential oils used in aromatherapy for stress relief may reduce the risk of cardiovascular disease [4, 5]. The reason is that short-term exposure to fragrance has a beneficial effect on heart rate and blood pressure [6, 7].

Previous studies have shown that some fragrances have inhibitory effects on brain and autonomic nervous functions [7, 8]. Haze et al. [9] reported that inhalation of pepper oil induced an increase in plasma adrenaline concentration compared to the resting state, while inhalation of rose oil caused a decrease in adrenaline concentration, and they concluded that inhalation of essential oils may modulate sympathetic activity.

As a conventional method for fragrance inhalation, a piece of cotton impregnated with diluted essential oil is placed under a subject’s nose to allow inhalation of the fragrance during ordinary breathing [9–11]. However, there is no appropriate experimental system to accurately and quantitatively evaluate the effects of sustained odor stimulations in humans. The lack of such experimental systems makes it dificult to investigate the time-dependent physiological effects of chemical composition variations in essential oils, and as a result the mechanisms of therapeutic effects remain unclear.

In this study, we aimed to develop a novel method to quantitatively evaluate the effect of odor stimulation on cardiorespiratory functions over time, and to examine the potential usefulness of clinical aromatherapy. In order to measure how much odor subjects have inhaled, respiratory and metabolic data were recorded using an automatic breath-by-breath respiratory gas analyzing system during the experiments, and two experimental step protocols under resting condition in a sitting or a supine position were performed.

## Methods

### Ethical statements

Prior to participation in this study, all subjects provided written informed consent after all the procedures and potential risks were explained. All experimental procedures and protocols conformed to the Declaration of Helsinki and were approved by the Human Subjects Committee of Morinomiya University of Medical Sciences (No. 2014–075).

### Experimental protocol and instrumentations and measurements

Eighteen subjects were assigned to participate in one of two protocols under resting condition. In protocol 1, nine subjects (7 men and 2 women) aged 26 ± 2 (SD) years, weighing 57 ± 10 kg and 162 ± 6 cm tall participated in the experiment with sitting position. In protocol 2, nine subjects (7 men and 2 women) aged 22 ± 3 (SD) years, weighing 58 ± 8 kg and 167 ± 6 cm participated in the experiment with supine position.

All subjects had no known cardiovascular or pulmonary disorders, had no history of head injury, and were not taking any prescribed medication known to influence systemic or cerebrovascular function. Prior to the experiment and after giving informed consent, each subject visited the laboratory to familiarize with the techniques and procedures. Subjects were requested to abstain from caffeine-containing beverages for 12 h, and strenuous physical activity and alcohol for at least 24 h before the day of the experiment.

Experiments were performed in a temperature-controlled laboratory (24 ± 1°C). After instruments were connected, the subjects rested in sitting or supine position while breathing room air through a face mask. To examine the effects of inhalation of sweet marjoram essential oil on blood pressure, heart rate and respiratory responses under resting condition, each subject was tested while inhaling air containing sweet marjoram essential oil (fragrant inhalation condition) and while inhaling air containing no essential oil (control condition).

Sweet marjoram essential oil (*Origanum majorana* oil; Mont Saint Michel Aroma Laboratory, Osaka, Japan) was diluted to a concentration of 1% in water (essential oil: water = 0.2 ml: 20 ml) and diffused using an ultrasonic aroma diffuser (DOSHISHA DAM-1101, Doshisha Corporation, Osaka, Japan) in a sealed acrylic box (60 cm x 60 cm x 60 cm) with two drain hoses. A fragrance by merging the water and essential oil was released by the ultrasonic diffuser into the air in the box at the rate of 0.27 mL / min. In addition, air flow of 5 L / min was introduced into the box through a drain hose, and accurately controlled using a gas regulator with high precision film flow meter (SF-1U; Sansyo, Tokyo, Japan). Therefore, it is expected that the essential oil was diluted with air to 5.4 x 10^−4^ ml / L (0.27 x 1 x 10^−2^ / 5). Air containing a constant concentration of essential oil was collected into 200-L Douglas bag. By repeating the same procedure, we succeeded to deliver odor stimulation at a constant level.

Protocol 1: After resting for 2 min in a sitting position breathing room air, the subject inhaled either air containing no essential oil (control condition) or air containing sweet marjoram essential oil (fragrance inhalation condition) from the Douglas bag for 6 min. For experimental trials, beat-to-beat heart rate (HR) and arterial blood pressure (BP), as well as breath-by-breath respiratory variables were recorded continuously. The order of the tests (control condition and fragrance inhalation condition) was randomized in each subject, with an interposing interval of at least 20 min.

Protocol 2: After resting for 5 min in a supine position breathing room air, the subject inhaled air containing sweet marjoram essential oil from the Douglas bag for 10 min, and then recovered for 10 min breathing room air. During the trial, beat-to-beat HR and BP, as well as breath-by-breath respiratory variables were recorded continuously.

After each experimental trial (control or fragrant inhalation condition) in both protocols, the subject was asked to rate valence (0: unpleasant, to 9: pleasant) and arousal (0: relaxing, to 9: stimulating) [12]. The rating scales were placed in front of the subject.

**Fig 1:**
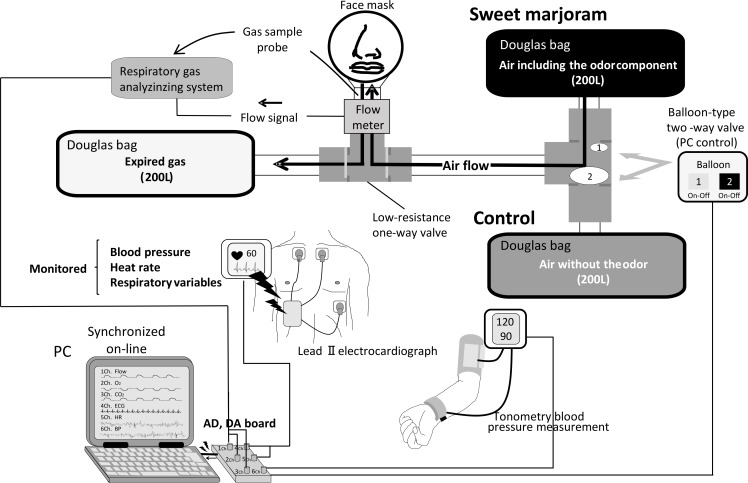
Experimental set up.

Signals from a respiratory gas analyzer (ARCO2000-MET, Arcosystem, Chiba, Japan) and an electrocardiograph (BSM-7201, Nihon Kohden, Tokyo, Japan) were synchronized on-line using a personal computer, and continuously displayed during all experiments. Oxygen and CO_2_ measurements were calibrated using standard gases of known concentrations before each test. HR was monitored using a lead II electrocardiograph. BP was monitored by tonometric blood pressure measurement (BP-608 Evolution II; Omron-Colin, Tokyo, Japan). Ventilatory responses were measured using a non-rebreathing open circuit apparatus (Model 8250; Hans Rudolf). The subject breathed through a face mask attached to a low-resistance one-way valve with a flow meter. The valve mechanism allowed the subject to inspire room air or a selected gas mixture with or without sweet marjoram essential oil from a 200-L Douglas bag. To avoid diffusion of aroma into the room, all expired gases was collected through a one-way valve with the hose attached to another Douglas bag (Fig 1). The total apparatus dead space was 200 ml. Respiratory and metabolic data during the experiments were recorded using an automatic breath-by-breath respiratory gas analyzing system. We digitized expired flow, CO_2_ and O_2_ concentrations, and derived tidal volume (V_T_), respiratory rate (RR), minute ventilation (V_E_), and end-tidal partial pressure of CO_2_ (P_ETCO2_). Flow signals were computed to single breath data, and matched to gas concentrations identified as single breaths using P_ETCO2_, after correcting for the time lag (350 ms) in gas concentration measurements. The corresponding O_2_ uptake and CO_2_ output for each breath were calculated from inspired–expired gas concentration differences, and by expired ventilation, with inspired ventilation being calculated by N_2_ correction. During each protocol, HR, BP, V_E_, P_ETO2_ and P_ETCO2_ were recorded continuously at 200 Hz.

### Data analysis

Beat-to-beat mean arterial pressure (MAP) was obtained from each waveform. Each steady-state cardiorespiratory data was obtained by averaging the respective data for the last 2 min. Dynamic BP response data at the start of odor stimulation approximated a first-order exponential model including lag time, and was quantified using Equation 1.

$$BP\left(t\right)=BP\left(0\right)+G_{u}•\left[1-\exp\left(-\frac{t-L_{u}}{\tau_{u}}\right)\right]$$

Where G_u_, τ_u_, and L_u_ represent the steady-state gain, time constant, and lag time, respectively.

Depending on the purpose of the comparison, a one-way repeated measure anaysis of variance (ANOVA) was conducted on each response variable using individual subject mean data. The mean data of each cardiorespiratory variable among the time segments for protocol 2 were analyzed by comparison with pre-inhalation data using the Tukey test. All data are presented as mean ± SD, and significance for all two-tailed tests was set at *P* < 0.05.

## Results

### Protocol 1

**Fig 2:**
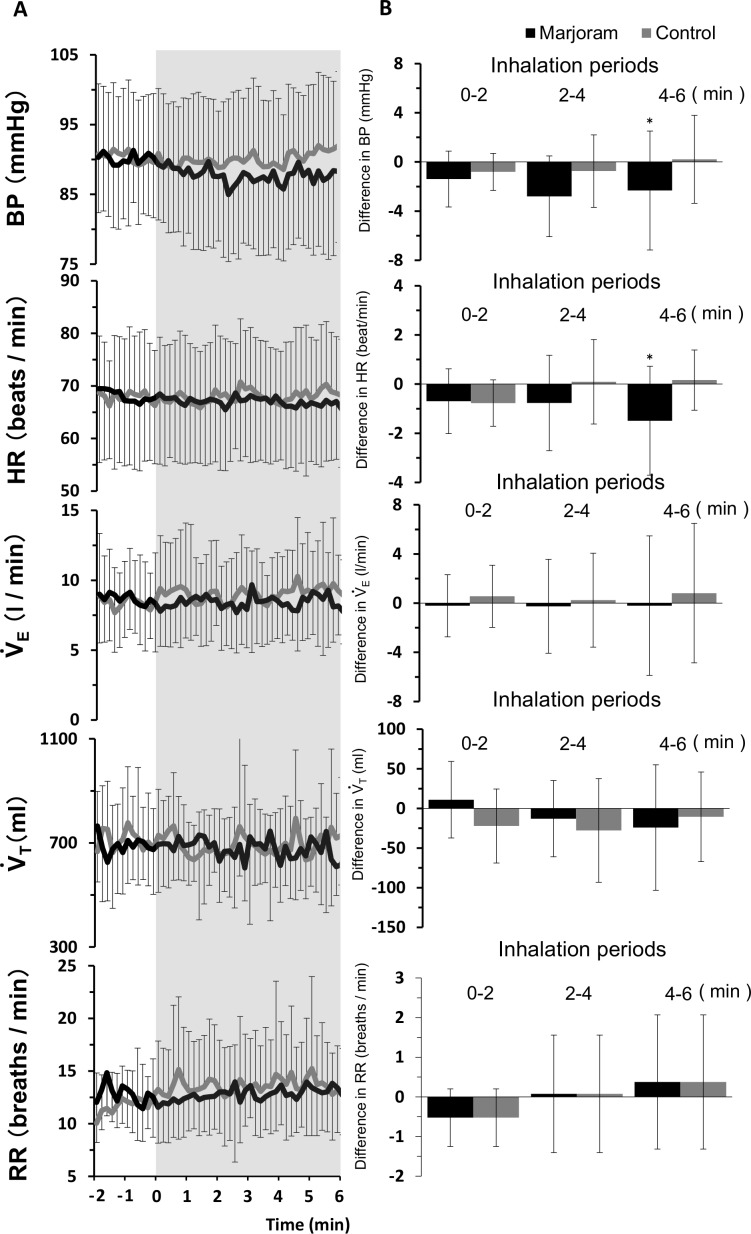
Cardiorespiratory variables to fragrance stimulation by inhaling sweet marjoram essential oil. Panel A: Time courses of cardiorespiratory variables to fragrance stimulation by inhaling sweet marjoram essential oil. Panel B: Absolute difference relative to baseline in each variable data for each of the three separate 2-min duration (0–2, 2–4 and 4–6 min). * P < 0.05 vs. the control condition.

Table 1 summarizes the steady-state cardiorespiratory baseline data obtained by averaging the last 2 min of the pre-inhalation baseline condition. There were no significant differences between the two conditions in all the parameters. The time courses of mean BP, HR, minute ventilation (V_E_), tidal volume and respiratory rate to odor stimulation by inhaling sweet marjoram essential oil are shown in Fig 2A. The changes relative to baseline in each variable data for each of the three separate 2-min durations (0–2, 2–4 and 4–6 min) were computed for the fragrance inhalation and control conditions (Fig 2B).

**Table 1:** Summary for the steady-state cardiorespiratory baseline data obtained by averaging the last 2 min of the pre-trial baseline condition in protocol 1.

Protocol 1 (n = 9)	Pre-inhalation Baseline					
	Control			Sweet marjoram		
BP, mmHg	90.3	±	9.9	90.2	±	8.8
HR, beats / min	67.9	±	11.5	68.1	±	13.5
V_E_, l / min	8.3	±	3.3	8.7	±	3.2
V_T_, ml	711	±	192	690	±	177
RR, breaths / min	11.7	±	3.6	12.8	±	3.6
V_O2_, ml / min	295	±	124	307	±	119
V_CO2_, ml / min	250	±	102	257	±	92

Vaules are means ± SD. BP, blood pressure; HR, heart rate; VE, minute ventilation; VT, tidal volume; RR, respiratory rate; VO2, oxygen consumption; VCO2, carbon dioxide production.

Remarkable changes in blood pressure and heart rate during inhalation of sweet marjoram essential oil were observed, but no changes in those parameters were observed in the control conditions. During fragrance inhalation for 6 min, the subject's minute ventilation was 8.4 ± 2.5 L / min. Therefore, we predicted that the subject inhaled 4.5 x 10^−3^ ml / min (5.4 x 10^−4^ × 8.4) of the essential oil. ANOVA revealed that fragrance inhalation significantly decreased BP (F_(1, 8)_ = 5.769, *p* = 0.043) and HR (F_(1, 8)_ = 8.623, *p* = 0.019) compared to control condition for the last 2 min. No significant differences in respiratory variables were detected between control and fragrance inhalation conditions (Fig 2A and 2B).

### Protocol 2

**Fig 3:**
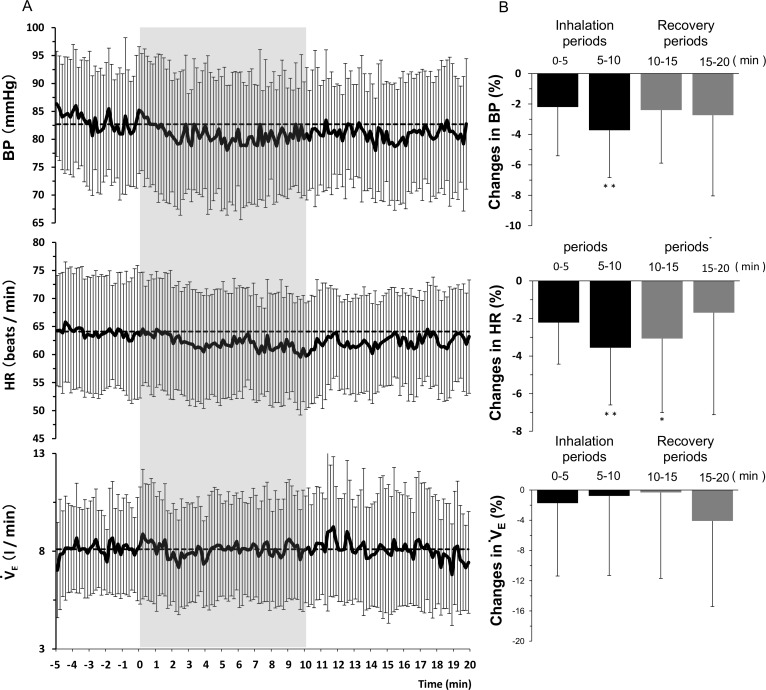
Cardiorespiratory variables during fragrance stimulation by inhalation sweet marjoram essential oil and post-inhalation recovery. Panel A: Time courses of mean BP, HR and VE to odor stimulation by inhaling sweet marjoram essential oil. Panel B: Percentage change from baseline in each variable data for each of the four separate 5-min duration (0–5, 5–10, 10–15 and15-20 min). * P < 0.05 and ** P < 0.01 vs. pre-inhalation baseline value.

The time courses of mean BP, HR and VE to odor stimulation by inhaling sweet marjoram essential oil are shown in Fig 3A. The changes relative to baseline in each variable data for each of the four separate 5-min durations (0–5, 5–10, 10–15, 15–20 min) were computed (Fig 3B). During inhalation of sweet marjoram essential oil, a gradual decrease in mean BP and HR were observed. The fragrance-induced hypotensive and bradycardic effects were attenuated during post-inhalation period. Similar change in VE was not observed. During the fragrance inhalation for 10 min, the subject's minute ventilation was 8.1 ± 2.5 L / min. Therefore, we predicted that the subject inhaled 4.4 x 10^−3^ mL / min (5.4 x 10^−4^ × 8.1) of the essential oil. Table 2 summarizes the cardiorespiratory data obtained by averaging the five separate 5 min during experimental trials. ANOVA revealed that a significant main effect of time observed for BP (F_(2, 16)_ = 7.323, p = 0.006) and HR (F_(2, 16)_ = 9.503, p = 0.002). Furthermore, BP and HR were significantly lower at 5–10 min than the pre-inhalation baseline values, indicating the time-dependent progression of depressor and bradycardic effects. During inhalation of sweet marjoram essential oil, blood pressure was—3.7 ± 3.1% lower (- 3.0 ± 2.6 mmHg, p = 0.004) while heart rate was—3.6 ± 3.0% lower (- 2.3 ± 1.9 beats / min, p = 0.008) than the pre-inhalation baseline values (Fig 3B). In addition, the siginificant fragrance-induced bradycardic effect lasted at least 5 min during post-inhalation period (- 3.1 ± 3.9% vs. pre-inhalation baseline value, p = 0.012).

**Table 2:** Summary for cardiorespiratory data obtained by averaging the five separate 5 min during experimental trials in protocol 2.

Protocol 2 (n = 9)	Pre-Inhalation Baseline (5 min)	Inhalation Sweet majoram		Post-Inhalation Recovery	
		0–5 min	5–10 min	10–15 min	15–20 min
BP, mmHg	82.7 ± 10.3	81.1 ± 11.4	79.8 ± 11.1**	80.7 ± 10.3	80.4 ± 10.6
HR, beats / min	64.1 ± 12.4	62.6 ± 5.0*	61.7 ± 9.6**	61.8 ± 4.9*	62.7 ± 8.9
V_E_, l / min	8.1 ± 1.1	8.0 ± 1.1	8.1 ± 1.1	8.2 ± 1.3	7.9 ± 1.2
V_T_, ml	715 ± 105	749 ± 111	750 ± 108	743 ± 110	765 ± 125
RR, breaths / min	12.5 ± 2.8	12.3 ± 3.5	12.4 ± 3.3	12.0 ± 2.3	11.6 ± 2.5
V_O2_, ml / min	274 ± 41	283 ± 49	276 ± 43	256 ± 48	270 ± 43
V_CO2_, ml / min	226 ± 35	226 ± 38	230 ± 38	228 ± 40	226 ± 39

Vaules are means ± SD., significantly different from pre-Inhalation baseline data.

* P < 0.05

** P < 0.01

**Fig 4:**
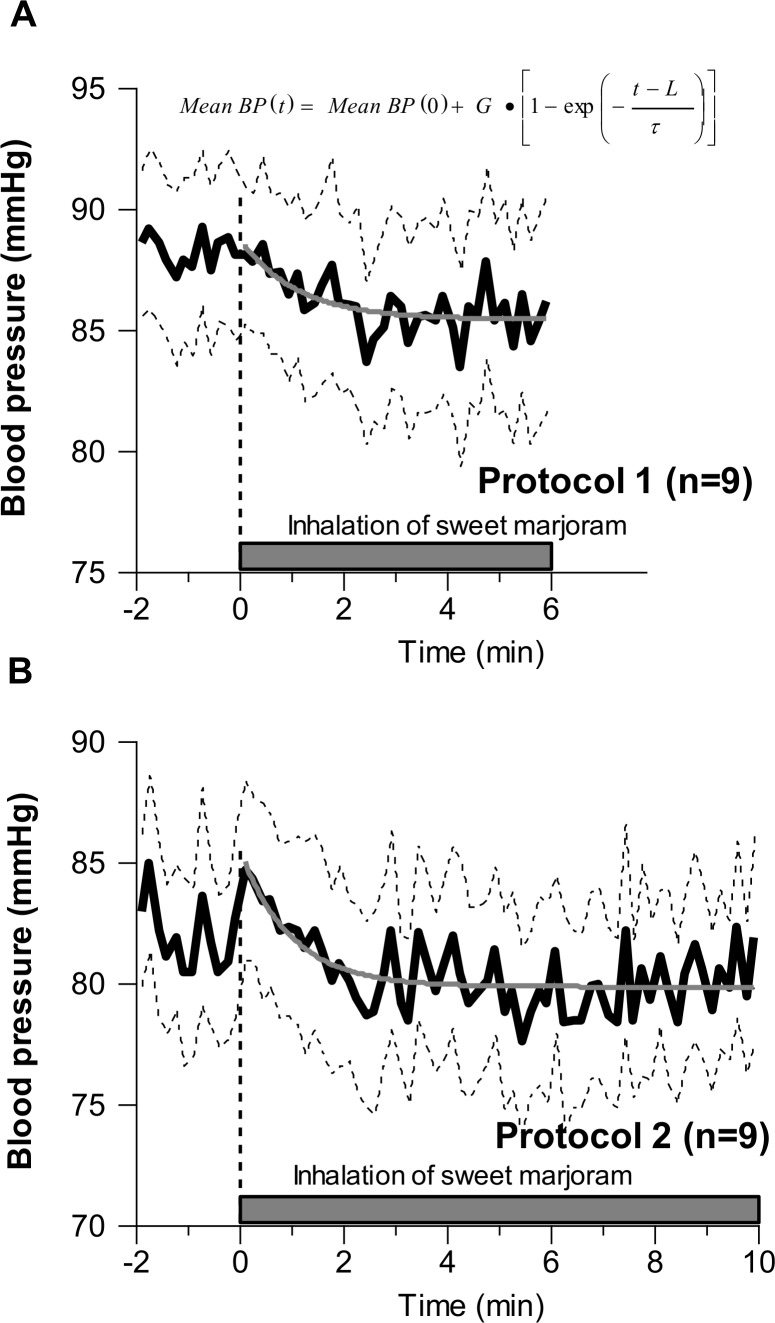
Time courses of mean BP response to a one step odor stimulation by inhaling sweet marjoram essential oil. Panel A: Protocol 1; The averaged regression line was $BP\left(t\right)=90.3-2.9•\left[1-\exp\left(-\frac{t-4.6}{48.4}\right)\right]$. Panel B: Protocol 2; The averaged regression line was $BP\left(t\right)=84.4-4.5•\left[1-\exp\left(-\frac{t-12.7}{58.8}\right)\right]$. Solid lines, means; dashed lines, means±SE.

Fig 4 shows the time courses of mean BP response to odor stimulation by inhaling sweet marjoram essential oil in protocol 1 and 2. For both protocols, the mean BP response at the start of odor stimulation was approximated by a first-order exponential model. The coefficients were estimated as follows: BP (0); 90.3 mmHg, gain (G); - 2.9 mmHg, lag time (L); 4.6 sec, time constant (τ); 48.4 sec in protocol 1. In protocol 2, the coefficients were estimated as follows: BP (0); 84.4 mmHg, gain (G); - 4.5 mmHg, lag time (L); 12.7 sec, time constant (τ); 58.8 sec.

**Fig 5:**
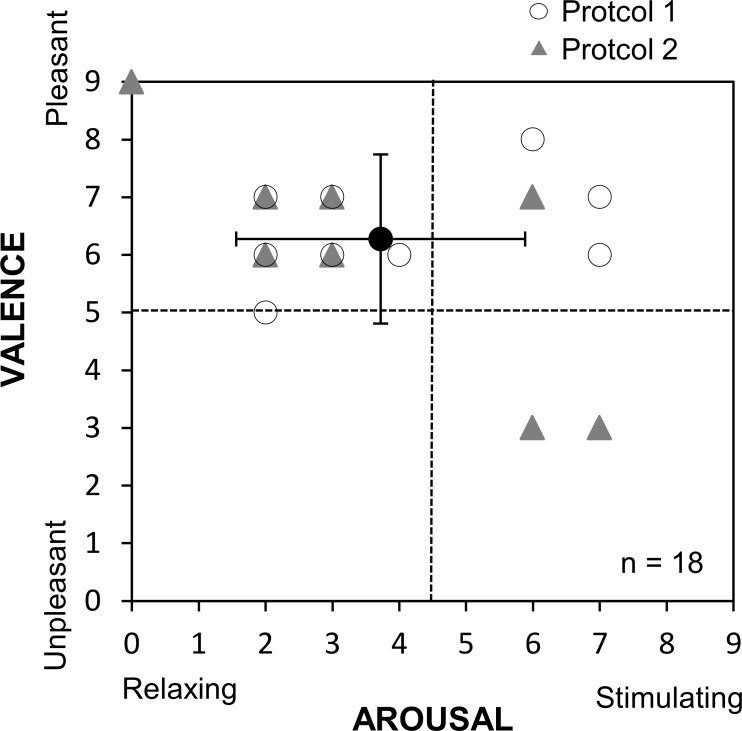
Bi-dimensional representation of the arousal and valence ratings of odor stimulation by inhaling sweet marjoram essential oil.

Fig 5 shows bi-dimensional representation of the arousal and valence ratings of odor stimulation by inhaling sweet marjoram essential oil. The mean arousal and valence ratings in each protocol were 2.0 ± 1.9 and 7.3 ± 1.8 (protocol 1), and 3.4 ± 2.4 and 6.1 ± 2.0 (protocol 2), respectively. While inhaling the essential oil, many subjects felt pleasantly relaxed or comfortable.

## Discussion

Using the conventional method of fragrance inhalation by presenting essential oil-impregnated cotton or paper under the nose [7, 9–11], it is difficult to accurately and quantitatively evaluate the dynamic behavior of the cardiorespiratory system against smell perturbations that vary constantly. In this study, we developed a new method using step response for the first time in humans to accurately evaluate the dynamic control of the cardiorespiratory system during the course of inhalation of essential oil. Furthermore, we showed the relation between the BP / HR change, and amount of inhaled odor (4.5 x 10^−3^ ml / min in protocol 1, 4.4 x 10^−3^ ml / min in protocol 2), the odor concentration and duration of inhaling odor, in addition to time-dependent progression of depressor and bradycardic effects during sweet marjoram fragrance inhalation.

Several studies have documented that some fragrances have inhibitory effects on autonomic nervous functions [2, 4, 9]. For example, Haze et al. [9] reported that inhalation of pepper oil induced a 1.7-fold increase in plasma adrenaline concentration compared to the resting state, while inhalation of rose oil caused a 30% decrease in adrenaline concentration. They also demonstrated the effect of fragrance inhalation on sympathetic activity in healthy human subjects using power spectral analysis of heart rate variability and blood pressure fluctuations. Therefore, our results suggest that inhalation of sweet marjoram essential oil decreases sympathetic nerve activity, and may induce the time-dependent progression of depressor and bradycardic effects.

In the present study, although precise mechanisms underlying physiological changes during fragrance stimulation are not yet clear, both physiological and psychological mechanisms can be involved in the effects of fragrance inhalation on biological reaction. For example, previously, Heuberger et al. [13] demonstrated that changes in autonomic nervous system parameters and self-evaluation were in part related to subjective evaluation of the odor, and suggested that psychological mechanisms may be involved in the autonomic and cardiovascular effects. For the current study using topographic mapping of electroencephalogram power, Sayorwan et al. [14] showed the evidence that the distribution of alpha brainwave activity, autonomic nervous system response, and mode states were affected by fragrance inhalation (lavender oil). Furthermore, in animal study, Tanida et al. [15], demonstrated that olfactory stimulation with scent of lavender oil affects autonomic neurotransmission and reduces blood pressure through the central histaminergic nervous system and the hypothalamic suprachiasmatic nucleus. We found the fact that many subjects felt pleasantly relaxed or comfortable, while inhaling the essential oil. Therefore, central brain olfactory sensing mechanisms may be involved in BP and HR change observed in this study.

On the other hand, contrary to this central brain olfactory sensing mechanisms, results from previous studies indicate that regulation of BP by inhalation of fragrance component has been caused by other sensing mechanisms without through nose. Umeno, et al. [16] demonstrated that direct Cedrol inhalation into the lower airway in laryngectomized subjects decrease BP. Pluznick et al. [17] also demonstrated that the major components of the olfactory signaling pathway are present in the kidney, where they play important functional roles in the regulation of renin release. In addition, Pluznick et al. [18] have recently reported that olfactory receptors are G protein-coupled receptors, and these receptors are expressed in smooth muscle cells of small resistance vessels to regulate blood pressure. Indeed, based on the observation that BP decreased gradually after the start of odor stimulation by inhaling essential oil, and the time constant was 48.4 and 58.8 sec, and thus relatively slow reaction in each protocol (Fig 4), the other peripheral regulators via a blood-borne route rather than central neurogenic regulation factors may be involved in the mechanism of modulation of sympathetic nerve activity. Further studies are necessary to elucidate how a central and the other sensing mechanisms are involved in BP and HR changes observed in the present study.

It is generally known that olfactory adaptation modifies the sensitivity to odor after prolonged exposure to the same odor; i.e., sensing of odor is strong at first and diminishes over time. Dalton et.al. reported that exposing individuals for a 2 week period to pleasant odor in their home environment produced diminished olfactory sensitivity, and the loss of sensitivity was measurable in situations outside the exposure context more than 6 h after any exposure [19]. In our experiment, since each subject smelled essential oil through a face mask for a short duration of 6 to 10 min, adaptation to odor was presumed to be negligible. However, further studies are required to examine whether adaptation to odors by long-term exposure or intermittent inhalation of fragrance may impact the effects of essential oil inhalation on cardiorespiratory response.

Recently, essential oils employed in aromatherapy are used increasingly for the improvement of quality of life as well as for the relief of stress and various symptoms in patients. Use of fragrant essential oils may reduce the risk of cardiovascular diseases. In addition, inhalation of sweet marjoram essential oil may be beneficial to correct sympathetic dysfunction associated with various lifestyle-related disorders such as hypertension and obesity.

## Conclusions

We established a novel approach to quantitatively and accurately evaluate the effects of quantitative odor stimulation on dynamic cardiorespiratory functions, and the duration of the effect. This methodological approach may be useful for scientific evaluation of aromatherapy as an approach to integrated medicine, and the mechanisms of action of physiological effects in fragrance compounds.
